# Crowdsourced Traffic Event Detection and Source Reputation Assessment Using Smart Contracts

**DOI:** 10.3390/s19153267

**Published:** 2019-07-25

**Authors:** Jernej Mihelj, Yuan Zhang, Andrej Kos, Urban Sedlar

**Affiliations:** 1 Faculty of Electrical Engineering, University of Ljubljana, 1000 Ljubljana, Slovenia; 2 Shandong Provincial Key Laboratory of Network Based Intelligent Computing, University of Jinan, Jinan 250022, China

**Keywords:** truth discovery, road traffic, event detection, reputation assessment, blockchain, smart contract

## Abstract

Real-time data about various traffic events and conditions—offences, accidents, dangerous driving, or dangerous road conditions—is crucial for safe and efficient transportation. Unlike roadside infrastructure data which are often limited in scope and quantity, crowdsensing approaches promise much broader and comprehensive coverage of traffic events. However, to ensure safe and efficient traffic operation, assessing trustworthiness of crowdsourced data is of crucial importance; this also includes detection of intentional or unintentional manipulation, deception, and spamming. In this paper, we design and demonstrate a road traffic event detection and source reputation assessment system for unreliable data sources. Special care is taken to adapt the system for operation in decentralized mode, using smart contracts on a Turing-complete blockchain platform, eliminating single authority over such systems and increasing resilience to institutional data manipulation. The proposed solution was evaluated using both a synthetic traffic event dataset and a dataset gathered from real users, using a traffic event reporting mobile application in a professional driving simulator used for driver training. The results show the proposed system can accurately detect a range of manipulative and misreporting behaviors, and quickly converges to the final trust score even in a resource-constrained environment of a blockchain platform virtual machine.

## 1. Introduction

Road transport is a cornerstone of modern society; despite many alternative modes of transport, vehicular traffic remains prevalent for personal mobility. Recently, there have been huge advances in the field of Intelligent Transport Systems (ITS) to make vehicular traffic safer, more efficient, and more user-friendly; such systems rely heavily on up-to-date information about traffic and road conditions.

Currently, traffic and road information acquisition is based on two principal models. Firstly, it can be sourced from roadside infrastructure (induction loops, surveillance cameras, speed cameras, Radio Frequency Identification (RFID) tags, etc.); such sources typically produce small amounts of high-quality data. Secondly, it can leverage crowdsensing, by pooling large quantities of lower-quality data, and then applying statistical modeling techniques to clean such data. Many providers use this approach, leveraging mobile terminals with Global Navigation Satellite System (GNSS) support and mobile apps to submit anonymized or pseudonymized data points of people’s location.

Another trend in the making is that of semi-autonomous and autonomous vehicles, which will—at least in the beginning—coexist side-by-side with manned vehicles [1]. In the future, it is expected that autonomous vehicles will rely not only on their own sensed data, but also on the data gathered by other vehicles and infrastructure. Data exchange between vehicles (Vehicle-to-Vehicle, V2V), infrastructure (V2I), and pedestrians (V2P) will become one of the key enablers of Cooperative Intelligent Transport Systems (C-ITS) and an important driver for autonomous traffic. With the increasing number of systems and users relying on such data, it will become of crucial importance to be able to assess its trustworthiness and detect intentional or unintentional manipulation, deception, spamming, and spoofing to ensure general safety and efficient operation. A robust event detection mechanism for crowdsourced data is, thus, an essential element for providing the stability and reliability of all the systems depending on it.

In mixed traffic systems, various data sources with different sensing capabilities and different security and trust levels will coexist. On one hand, offences such as speeding or driving through a red light are well defined, and these can already be detected within lowest margin of error using dedicated traffic cameras and radars. As the next evolutionary step, the increasing number of sensor-equipped connected cars will further extend the reach and increase the granularity of traffic event detection. However, more complex events, such as dangerous or aggressive overtaking, mobile phone use, or illegal parking, will still be difficult to detect reliably with sensors alone; people are currently the best pattern recognizers and reporters of such situations—if properly incentivized.

Of course, properly incentivizing people to provide more high-quality data plays an extremely important role in crowdsensing, where we face the data quality vs. data quantity dilemma [2]. This has been an area of active research, and today, many reward mechanisms are known and used, based either on monetary reward schemes, on users’ reputation, or a combination of both. However, in today’s world, the concerns about people’s privacy are a strong counterforce to incentive mechanisms.

Today, millions of people already make their decisions based on the crowdsensed traffic data, e.g., when using navigation applications with real-time road and traffic condition updates. However, it has been shown that even such applications are prone to attacks. Therefore, several mechanisms for effective truth discovery in traffic systems have been designed to solve this [3,4,5]. Traffic events and conditions can be classified into several categories, depending on measurement and sensing equipment and their capabilities, and the purpose of data gathering. As defined in C-ITS Day 1 applications list, the basic traffic event set consists of accidents, traffic jams, dangerous weather conditions, and obstacles in the road. Additionally, driving through a red light, speeding, wrong way driving, and careless driving are considered to be included in reporting application. Thus, truth discovery mechanisms will differ based on the optimal balance needed between accuracy and computational complexity.

The concerns people have about privacy in centralized systems should be addressed without compromising general system reliability and robustness. To overcome current limitations emerging from centralized crowdsensing solutions, decentralized computing platforms could be used.

In this paper, we focus on a mechanism for detection of traffic events from user- or machine-originated crowdsourced data. Special care is taken to adapt the system for operation in decentralized mode on a Turing-complete blockchain platform, eliminating single authority over such systems and increasing resilience to institutional data manipulation. In addition, we aimed to also respect the privacy of the users, which is especially important when using public ledger technology with all data in plain sight. Special attention was given to the evaluation of a proposed solution, using both a synthetic traffic event dataset and a dataset gathered from real users, using a traffic event reporting mobile application in a professional driving simulator.

The rest of the paper is organized as follows. Related work is described in Section 2. In Section 3, system design, event detection, and source reputation assessment mechanisms are presented. Section 4 describes solution verification setup, followed by details about centralized and decentralized implementation. In Section 5 we provide a conclusion and also introduce our future work.

## 2. Related Work

Today, crowdsensing systems often act as an intermediate system between users that provide data, and data consumers on the other side. Including people in such systems increases the probability of spoofing, malicious user behavior [6]. Therefore, assessing the trustworthiness of users can prevent potential malicious behavior of such users, or at least lower the consequences.

Distributed systems have gained attention in communications, especially in vehicular networks, where Vehicular Ad-Hoc Networks (VANETs) have been at the core of research during the last decade. Even though such systems operate in a distributed manner, data collection and processing are still mainly done in centralized data processing centres [7].

A centralized approach is in stark contrast to the real picture, where, considering the traffic as individual vehicles and other participants, entities in vehicular traffic form a heavily distributed system. Connecting to the internet or to mobile ad-hoc networks (MANET), entities in reality form a dynamic distributed Cyber-Physical System (CPS) [8], which remains the same for Cyber-Physical-Social Systems (CPSS) where people and devices are part of the same system and operate side-by-side [9,10]. On the other hand, distributed operation of such dynamic systems has been difficult to achieve until this last decade, when concepts such as fog and edge computing emerged. While communication problems have been successfully addressed, there are still open problems yet to be solved on computational layers [11]. In addition to synchronization and distributed computation issues, open-up environments where everybody can participate revealed trust-related problems that have to be addressed.

Apart from distributed databases and data storage, implementation of Distributed Ledger Technologies (DLT) provides platforms for distributed consensus. DLT technologies, with blockchain as the most prominent form, show that consensus can be achieved among entities that do not necessarily trust each other, even in a highly distributed system [12]. Incorporating cryptographic functions into decentralized peer-to-peer networks, in a way that peers can achieve consensus, is providing a platform for decentralized applications that require trust, without relying on a trustable third party organizations [13]. Moreover, blockchain is currently the only mature-enough technology that leverages trustless and persmissionless operation of distributed systems, while at the same time provides resistance to censorship [14].

Besides mechanisms for achieving consensus, tamper-proof storage, and higher resilience due to distributed operation, blockchain platforms can also leverage public key identification. In the real world, governmental services require government-issued identities, while web applications can, for example, rely on social network identities or personal email addresses. In the blockchain world, public addresses or identities derived from addresses are used for identification purposes—e.g., Ethereum users are identified via their public addresses, derived from the private part of private-public cryptographic key pair. Identity management frameworks and platforms are crucial for smart city applications [15], where interconnection of people, devices, and services is crucial for effective and reliable city operations. Researchers in [16] proposed a secure pseudo-identity based identification service for smart cities. Moreover, blockchain can be used for both identification and authentication purposes, as shown in [17]. Despite such contributions that provide us platforms for device identification management, they do not extend to human participants that provide the foundation and the reason for the existence of smart cities [18,19].

Additionally, traffic infrastructure research projects such as C-roads [20] already propose interoperability processes based on hierarchical public key infrastructure for vehicle identity and message signing. This has the advantage of total vehicle traceability, which is a solid foundation for governmental registration. However, such a central authority system would make it extremely hard and time-consuming to provide a seamless and extensible layer of identities for other devices, managed by the owner. Meanwhile, our proposed approach can span across government-issued identities, as well as self-issued identities, in a decentralized manner.

Smart contracts (SC)—applications running in a decentralized manner on the DLT Virtual Machine, provided by a DLT platform—can be seen as autonomous agents responding to messages sent by other users in the network. Decentralized execution on multiple nodes across a DLT network provides resilience to attacks, while on the other hand, it lowers the efficiency and execution speed of such distributed systems [13,21,22]. Adding people to device-only Internet of Things (IoT) systems potentially adds non-trustworthy entities. In such environments, reaching consensus in a decentralized way is crucial for many emerging applications. Relying on SCs [23], not only distributed data storage and trustless operation can be established, but the system can at least be partially automated.

Relying on the above-mentioned properties of DLT, crowdsourcing and crowdsensing solutions have emerged. The most well-known applications running on DLT platforms today are cryptocurrencies. Platforms like Ethereum support custom tokens, which have also led to crowdsourcing platform projects. Regardless of openness of such solutions, monetary incentivizing mechanisms [24] are a natural fit for blockchain platforms. Even though blockchain platforms are based on verifiable chained data blocks that are visible to everyone in the network, privacy-oriented solutions have emerged in vehicular technology field [25]. Researchers in [26] proposed an incentive mechanism that uses a blockchain-based cryptocurrency as a secure way for user incentivization. Another interesting blockchain-based crowdsourcing framework is presented in [27], solving the requester–worker relationship by requiring the workers to make a time-locked deposit as a guarantee for their behavior.

Even though trust is expected to be of a greater concern in mixed human-device systems, only a few implementations currently address problems outside the device-only IoT environments [14,28]. However, blockchain, and especially SC-based solutions, currently present a promising technology for security- and privacy-related problems, both in device-only and mixed environments [29,30].

Especially in mobile and traffic crowdsensing, data quality also heavily depends on the location data quality. While smartphones and in-vehicle technology can provide sufficiently high accuracy location data, this also lowers users’’ location privacy. The accuracy-privacy trade-off problem is addressed in [24], which proposed a coalition strategy that provides a single identity data collection, while at the same time sharing the payoff between multiple involved users. Revealing location data, one can gather a comprehensive insight into one’s daily behavior by combining multiple data sources.

In addition to users’ privacy requirements, CPSs and CPSSs have to provide support for trustable operation of such systems. While trust between devices has already been addressed by many researchers, it is still a complex problem yet to be solved. Mimicking human-like relationships, social IoT addresses trust-related problems in device-only IoT environments [31,32]. However, in terms of crowdsensing and sensed data gathering and analysis, source reputation assessment plays an important role. Especially in open-up systems, where people and devices are not a priori verified, every data source can potentially disrupt the system by providing noisy or intentionally corrupted data [33].

Therefore, truth discovery cannot be based solely on crowdsourced data. Most prevalent techniques for truth discovery from crowded data are based on statistical inference. In traffic crowdsensing, Bayesian inference, expectation maximization, and majority voting are among the most popular [4].

Bayesian inference obtains the answer by computing posterior probability based on a priori known distribution. Results obtained using expectation maximization, which relies on the assumption that links users’ reliability and probability of the true result, are calculated iteratively. There is the major drawback for extensive usage in real situations. Using majority voting, the answer with the most votes is considered as computed truth. Besides them, mechanisms based on Gompartz function and fuzzy logic models are also widely used.

To the best of our knowledge, existing solutions mostly cover trust management in device-only IoT and CPS environments. Vehicular traffic event detection solutions are mostly limited to the implicitly collected user location data and data from road operators. Moreover, crowdsensing-focused solutions are generally centralized, which lowers the user privacy. We address these challenges in the following chapters.

## 3. Proposed Solution

### 3.1. Assumptions and System Design

We propose a smart-contract-based mechanism for truth discovery in a traffic event-reporting scheme. One of the requirements for the proposed solution is the support for heterogeneous data sources—vehicles and road infrastructure, as well as people using various reporting applications. Support for various data sources is crucial, since it increases the amount and the scope of the collected data. Thus, supporting multiple data sources can provide contextual data and allow the system to be extended with different types and grades of sensors. Thus, collected data will, in the next stage, be used for event detection, truth discovery, and iterative source reputation assessment.

Generally, the proposed solution is based on the inclusion of people and their perception of traffic events around them. Even similar events can be perceived differently, depending on the location, time, current traffic situation, and peoples’ current state and previous experience. In general, we cannot consider crowdsourced traffic reports as objective, but must take into account also the reporter’s perception of the events. Moreover, we should not expect that people will report all perceived events. In fact, not all events are even detected by people. Thus, when developing the event detection scheme, we tried to take into account also the reporting preferences and subjective event perception. Additionally, subjective perception is an important factor for source reputation assessment; thus, noisy reports should have much less negative impact on user reputation than misreporting.

The main goal was to design a robust event detection system, which can discover the truth from user-generated reports of traffic events, while at the same time preserve their privacy. Detected event data has to be open and auditable, thus they can be reused in other systems—e.g., traffic notification and alerting systems.

The system was designed for event detection on a set of reports from various sources. Due to different sensing capabilities that depend on source type, we aimed to keep the data requirements low. This resulted in a simplified data model, which supports event reporting from vehicles, infrastructure, and users via their own smart phones. All reports have to carry essential data, such as a timestamp, source type, and source identification if it cannot be obtained from the device ID, location data, or event type. Perceived consequences or the severity of the event is not a mandatory attribute, due to the fact that certain types of events cannot be sensed by all source types. Moreover, as mentioned above, events are perceived; therefore, their consequences or severity cannot be objectively measured.

**Figure 1:**
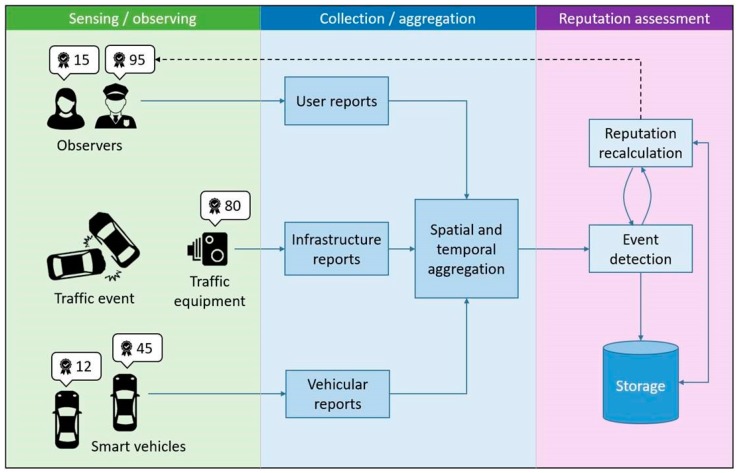
Event detection and source reputation assessment system.

The architecture of the proposed reporting and truth discovery system is modular, consisting of event aggregation, event detection, and source reputation modules as seen in Figure 1. Additionally, output from the event detection module can also serve as an input to external event notification and broadcasting services.

### 3.2. Data Model

Proposed solution is based on a simplified data gathering process, where users and devices provide traffic event observations to the event aggregation and detection system. Following the aims to design an open system in which data sources can freely contribute their observations, we aimed to eliminate the need for source management in terms of registration or a priori source verification. Therefore, source identification data is either attached to every event report or is gathered from report metadata, depending on implementation.

**Figure 2:**
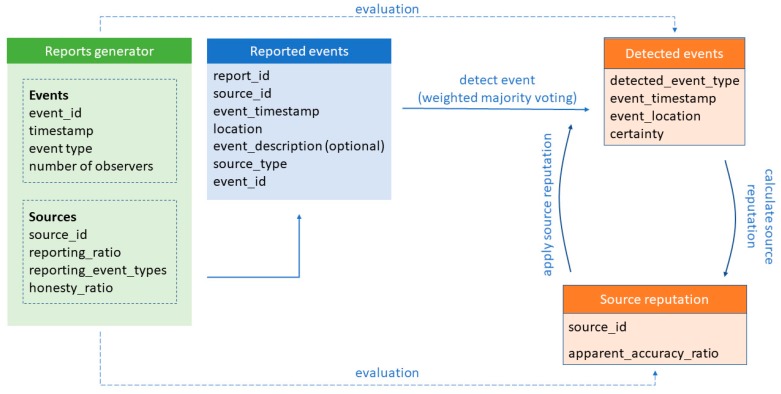
Event reporting and detection, and source reputation assessment data model.

To keep bandwidth and storage requirements low, data sources have to provide only essential data—event timestamp, detected event type, and location. Depending on implementation, observed event timestamp can also be obtained from the report metadata. However, it is recommended to include timestamp at the time when the event is detected, or at the time when the event report is being constructed and sent to the aggregation system (Figure 2). Location data is expected to be in the form of latitude and longitude value pairs. Depending on the reporting source, different location accuracy is expected and allowed—i.e., location of roadside equipment is well-defined—while vehicles and users’ smartphones report their location with a margin of error. As roadside equipment is considered as most trustworthy, their location is used as a reference for events reported by them and other sources nearby.

The event detection module operates over the limited set of reports, which consist of data representing the same event. Source identification data is not directly used in that phase—it is only used to acquire source reputation values that later serve as a weight in the event detection phase.

### 3.3. Event Detection and Source Reputation

We aimed to construct an event detection scheme that could run in a decentralized manner on a blockchain platform, which dictated several constraints. Smart contracts (SC)—applications that run on a DLT platform—have to be deterministic. That way, we can guarantee that execution of SC will return the same results, regardless of the node on which the contract is processed. For example, solidity, the de-facto programming language for SCs on the Ethereum platform, only supports deterministic functions.

Event reports are first grouped by location and timestamp. As reports are user- or device-generated and not sensed at the exact time the event occurs, location and timestamp may vary to some degree; thus, time and location windows are used to group reports for the same event. Reports belonging to the same event are then used for event detection.

Initially, all source types are considered as fair sources with high event reporting accuracy. These parameters are represented as a single metric—source reputation. Depending on source type, source reputation can be recalculated. Governmental and roadside equipment—mostly consisting of traffic cameras, speed cameras, traffic density detectors, and traffic lights sensors—have to be calibrated, and are typically maintained on a regular basis. Therefore, these types of data sources are considered as accurate, and are given a higher source reputation value. User reports, on the other side, are prone to mistakes and misreporting due to the human nature of perceiving events, instead of objectively observing them. Therefore, the users’ source reputation value is iteratively recalculated after every event report. The source reputation value consists of the number of reports and the ratio of correct reports. Combining these two parameters, the impact of sources with a lower number of reports is limited.

**Figure 3:**
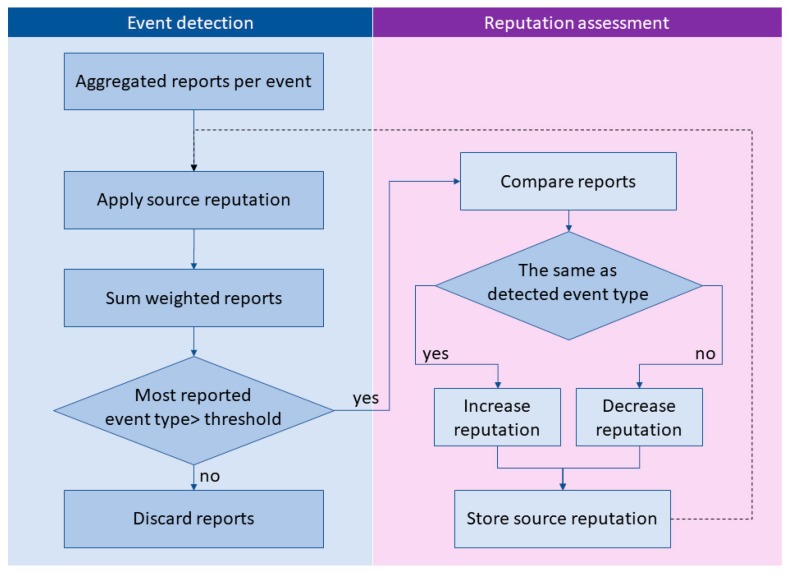
Event detection mechanism: Weighted majority voting.

Events are detected using the weighted majority voting mechanism (Figure 3), where source reputation values are used as weights. As all reports have to include the observed event type, reports are a priori classified into several categories—accidents, traffic jam, dangerous weather conditions, and obstacles on the road, as defined in C-ITS Day 1 services. Additionally, driving through a red light, speeding, wrong way driving, and careless driving are considered to be included in the reporting application. End users are encouraged to select the appropriate traffic event from the list, which helps to minimize the granularity of reports, as well as to provide better user experience. Reports per event are grouped and counted by reported event type. An event is successfully detected when the count of event type with most reports is greater than the threshold, currently set to two thirds of the number of all reports per event, but can be adjusted according to reporting accuracy of users. In general, for *m* misreports, *n* ≥ 2*m* + 1 reports are needed to provide resilience to such misreports.

In cases where reports do not converge towards the common event type, we cannot achieve the consensus. To reduce the number of such cases, we limited the number of possible event types from which the users choose when reporting the observed event. If reports still do not converge to the common value, which means that the event cannot be successfully detected, the source reputation assessment phase is skipped. Reports can be stored for future analysis or discarded to save storage capacity.

For driver alerting and notification purposes, only detected events are returned, without the data about event reporters. We aimed to keep the privacy of data sources as high as possible by such limited revealing of information.

## 4. Implementation and Verification

The proposed event detection mechanisms were tested on a set of well-defined simulated test cases, as well as data obtained from users. We constructed an initial set of traffic events that represent a number of possible traffic scenarios. Further, as we aimed to detect events from user reports, we built an event report generator.

**Figure 4:**
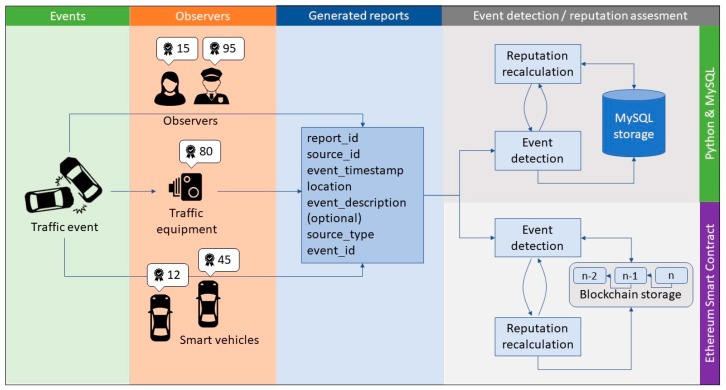
Verification of event detection and reputation assessment based on an artificially generated set of reports.

The implemented system can be divided into two main components (Figure 4). The first is used for event report generation. Traffic observers, vehicular and infrastructure data sources, and the initial event set are stored into static tables and serve as a source for the event generator module. According to scenarios, non-deterministic generated traffic event reports are stored in the MySQL relational database. These reports then stay unchanged for both relational database implementation, as well as for distributed implementation using SC.

The proposed event detection and source reputation assessment mechanism was first implemented using a relational database as a centralized solution. Centralized system architecture supports event report gathering and aggregation, and centralized event report storage. Stored events from relational database are then processed in the event detection module. All acquired data is stored, analyzed, and managed centrally.

The deterministic event detection mechanism is implemented using Python programming language on data acquired from the MySQL database. The event aggregation module acquires event reports from a relational database, and groups reports for the same events according to proposed schema—by comparing their timestamps and location. Aggregated reports are sent to the event detection module. Knowing the number of events, reported event types, and source type for every report, the truth discovery mechanism is applied to detect the event types that has most likely happened. Sources that reported (voted) for the event type that has been detected as most likely are given a rise in their reputation factor. Similarly, other sources’ reputation factors are lowered. Detected events, with accompanied probability that the detected event type is real, are stored in the relational database. Assessed source reputation values are stored and used as weights in future event detections. These results can be compared with the initial set of events.

### 4.1. Blockchain Implementation

The whole event detection and source reputation assessment system was also implemented in a distributed manner. We constructed the SC-based solution on the Ethereum platform, which provides additional robustness to the solution.

**Figure 5:**
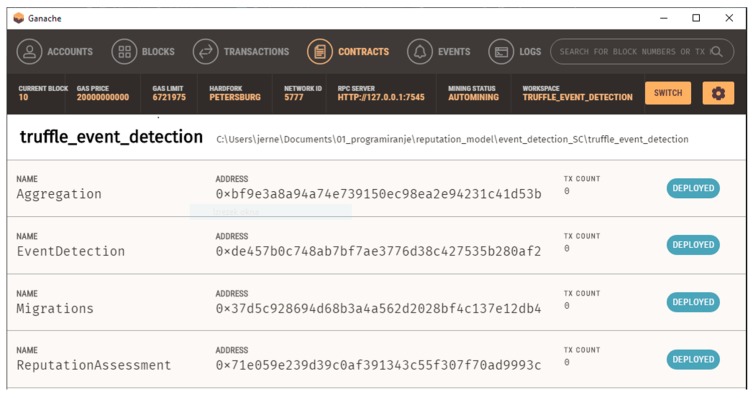
Ganache environment for local smart contract deployment and testing.

Compiled and deployed SCs are assigned unique public addresses that act as an interface for interaction with other entities on the network (Figure 5). As transactions are public, everyone can observe traffic between end users (data sources) and SCs in a way that reveals only pairs of nodes that communicate with each other. Data exchanged via transactions are encrypted, thus observers cannot access the content. However, observing transaction traffic, one can construct the network of interactions, which can lead to lowering user or device privacy.

**Algorithm 1:** Pseudocode of blockchain implementationreports = [list of traffic reports made by users, vehicles and infrastructure] events = [dictionary of reports belonging to the same event] reputation_weights = [dictionary of reputation values for each data source] function aggregate_reports(reports):    for each report_i_ in reports:        for each event_j_ in events:            if (report_i_.time <= event_j_.time + max_delta_t) and abs(report_i_.location - event_j_.location) <= max_distance):                add report_i_ to event_j_        if no match:            create new event and add report_i_ to it         if count of reports for event_j_ == 5:            detected_type = detect_event_type(event_j_)            if detected_type:                store detected_type                reputation_weight = assess_reputation(event_j_, detected_type)                store reputation_weight function detect_event_type(event):    get reputation_weights for all reports in event based on reporting data source     detected_event_types = []    for each report_i_ in event.reports:        detected_event_types.push(report_i_.event_type * report_i_.source.reputation_weight)     if max(detected_event_types) > threshold:        return max(detected_event_types) function assess_reputation(event, detected_event_type):    for each report_i_ in event.reports:        if report_i_.event_type == detected_event_type:            increase reputation_weight        else:            decrease reputation_weight aggregate_reports(reports)

The proposed event detection mechanism was implemented using the Truffle framework [34] on Ganache [35]—a local Ethereum-like blockchain platform. Using a local blockchain, we can observe and examine all transactions in the SC lifecycle, from deployment to operation state. Additionally, we can determine SC execution costs in “gas”, which is used as fees for mining nodes.

Implementation-wise, we developed the SCs in the Solidity language. Relatively simple validation was done using the built-in Ethereum accounts simulation. That way we were able to interact with the SCs was similar to the interactions to be expected in real-life implementation on public Ethereum blockchain. Due to the limitations of Ganache, we were not able to analyze transaction times; however, SC execution cost monitoring was supported, thus enabling us to analyze the SC operation.

The system was already designed to be modular and deterministic, which helped us separate event aggregation from detection. As events can be reported sporadically, as is expected due to human mobility and differences in observations and responding times, only aggregated event reports are sent to the detection module, which bring down the time needed for event detection phase.

While blockchain SC guarantees tamper-proof operation, publicly shared data could potentially lower user data privacy. Taking this into account, data management and variable scope has to be carefully decided. Limiting access to functions to only SC owner, and limiting functions visibility and scope, can greatly reduce potential data exposure. Following good practices, the system was split into aggregation, event detection, and reputation assessment modules, which, in distributed implementation on Ethereum, were implemented in separated smart contracts.

The proposed solution consisted of two main phases—event aggregation and event detection with reputation assessment. We aimed to provide robust event detection, while at the same time protecting the identity of the users. Thus, probably the most straightforward solution, in which user reports are stored on the blockchain and later processed, has to be discarded as it reveals user data. Moreover, storing event reports directly on the blockchain is relatively expensive, as it results in blockchain state changes that have to be verified by miners. Therefore, event reports are processed in-memory by the SC. While lowering execution and storage costs, it can also lower execution time. Only results of the weighted majority voting and source reputation are permanently stored on the blockchain.

**Figure 6:**
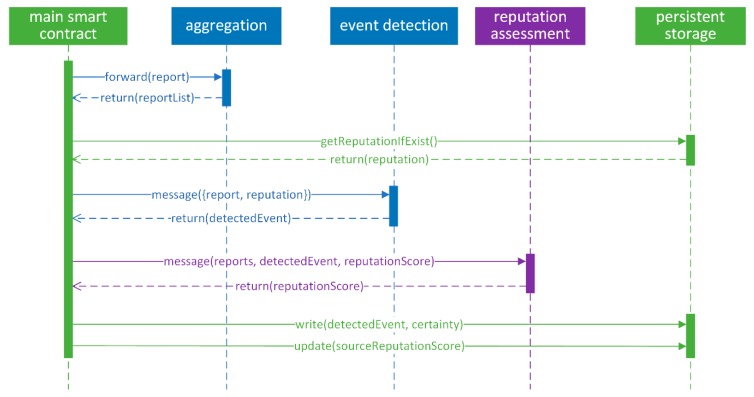
Interactions between smart contracts and persistent storage accessed by main SC.

Reports received by SC are stored as report objects in a structure similar to a hash table, with timestamps and source identificator combined used as keys to access the data. Due to limitations of blockchain platforms, transaction timestamps are likely to be unique; thus, we do not expect consequent or duplicated values. Separate arrays are used to keep the list of observers and indexes of stored reports, to allow us to access the reports. In the aggregation phase, as described in Algorithm 1, we loop through reports and group them by timestamp and location. Another auxiliary index is used to keep the record of reports belonging to the same event. That way, we keep the stored data untouched, which minimizes state changes, thus significantly lowering the computational expenses. Custom garbage collectors are used to clean-up auxiliary index arrays to prevent the uncontrolled growth that could lead to higher execution costs. Source reputation values are stored in key-value storage and are accessed using Ethereum platform-provided addresses. Thus, low probability for key duplication is expected. As all modules—event aggregation, event detection, and reputation assessment—work on in-memory data, only the main SC serves as a central manager to persistent data storage as shown in Figure 6.

The proposed mechanism is, thus, implemented separately, which provides us with a higher level of data privacy. In the event aggregation module, user identities can also be pseudonymized. As pseudonymized data is sent to the event detection module, less user data is revealed. In the event detection module, only reports that belong to the same event are processed, thus, no correlation between reports of the same user can be discovered. On the other hand, this limits the options for more sophisticated malicious behavior detection. However, modular implementation provides us options for further application upgrades.

### 4.2. Verification and Results

People involved in traffic are not just the observers of traffic and traffic events. They are a part of traffic, and their reactions to events and the environment around them can affect other people nearby. Thus, we cannot expect that they will objectively sense events around them. In fact, perception of traffic heavily depends on their personality, previous experience, their current mood, weather, the reason they are on the road, etc. In order to simulate and verify event detection from events reported by users and other sources, some assumptions were made.

The proposed solution was evaluated on set of artificially generated traffic events and reports related to them, as well as on user reports gathered from a web-based event reporting application. As the proposed solution was designed for event detection from various sources—infrastructure, vehicles, and users—we constructed an event report generator to build a static event report database that was used for evaluation. Roadside infrastructure reports are considered as accurate, however, with limited sensing capabilities; these reports are limited to red light and speeding offence detection. Vehicles are initially assigned an apparent accuracy ratio of 0.8. The user, as last source, cannot be considered as a fair and completely accurate source; therefore, we pay more attention to user reporting modeling.

The synthetic traffic event dataset was constructed with attention to covering all event types defined in proposed solution. In addition to events representing accidents, based on Slovenian Traffic Safety Agency reports, congestions and traffic jams, and dangerous and aggressive driving scenarios were also considered. Several events were strategically placed near schools and kindergartens, while others near arterial roads, on regional roads and highways. These locations serve as an input for event report generation.

We built an initial set of personas to model user sensing and reporting behavior. This set of personas serves as an input for the event reports generator. However, realistic user reporting models are hard to obtain without extensive user studies. Instead, we limited the number of possible event types that users can report. This is beneficial both for event detection system modeling, as well as for usability and user experience of end-user smartphone application. For every persona from the initial set, we constructed a scenario, which defines which events the selected user could detect.

**Figure 7:**
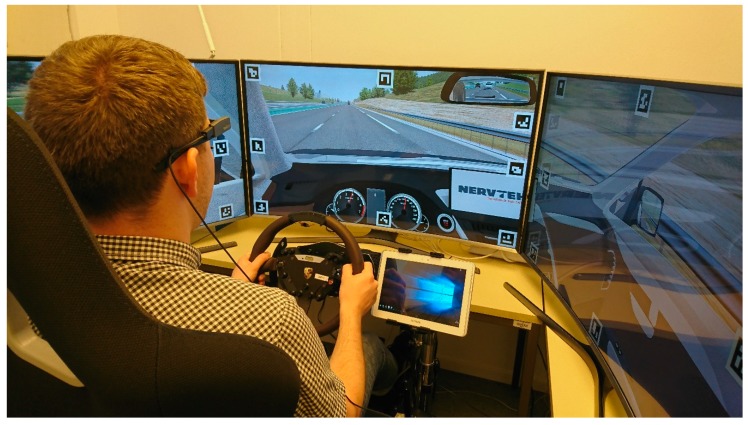
Obtaining user reporting behavior in professional driving simulator used for driver training.

To improve artificially generated personas, eight interviews were performed to obtain user’ reporting preferences. Moreover, their reporting behavior was evaluated in a driving simulator as seen in Figure 7. Five persons agreed with evaluation in driving simulator. All of them were tested on the same scenario, consisting of an initial few minutes of free drive and the driving on a looped road with various traffic events—broken down vehicle, animals at the side of the road, congestion due to one lane closing, and bad weather conditions—occurring at predefined locations. All users were asked to use a simple web-based traffic event and road conditions reporting application on their smartphones. Eye-tracking devices were used to monitor their reporting behavior (event types they were reporting, delay between event occurrence and report) in various traffic conditions and environments. Obtained data, combined with data gathered from questionnaires and interviews, were used to improve the artificially generated initial set of personas.

The rather small initial set of personas was expanded to the final set of 100 artificial users, and reporting parameters/preferences for every data source were varied. According to every persona’s event reporting ratio and apparent accuracy ratio (Table 1), simulated crowdsourced event reports were generated. Apparent accuracy is a combined metric that represents both user honesty and accuracy in event reporting ratio.

According to studies of human mobility, events and source mobility scenarios are roughly distributed during the week and time of the day to ensure that the number of event reports could follow the same patterns. In general, scenarios represent traffic events in dense and sparse traffic, during the day and night time, in city environments as well as on regional roads and highways. Event reports are stored in a relational database and are later used for verification purposes of both approaches. Additional sources are created to represent a wide range of users, ranging from totally trustworthy users that report every event they observe in a completely objective way, to malicious users that misreport every event they observe. Scenarios for personas are constructed in a way to distribute reports from all personas included between all events. Otherwise, voting groups—a group of users traveling along the same path at the same time—could occur. This could lead to wrong event detection, if a group of misreporting users is formed. Malicious users remain an important issue to be resolved. To some extent, malicious users are penalized by lowering their reputation value. However, some more advanced fraud detection techniques could possibly be implemented via external services. Both the initial set of events, as well as table of personas, are used to verify the proposed event detection mechanism and source reputation mechanism.

Implementing proposed event detection mechanisms, both traditionally using relational database, and in a decentralized manner on the Ethereum blockchain platform, gives us interesting insights about usability and real-life operation of such services. While computational complexity of event detection using weighted majority voting stays the same for both implementations, computational time and memory usage greatly differs between them.

The proposed solution was evaluated on the same set of event reports. In more traditional implementation using a relational database, we did not observe any limitations or lower performance. Otherwise, to implement proposed solution on the Ethereum platform, we needed to design and implement a customized storage solution from scratch. In the verification phase, this was identified as the biggest limitation, and also the part of the system that caused the most running costs.

The proposed solution was tested by continuously pushing event reports to the event detection and reputation system. Based on 400 events, 2524 reports were constructed using 100 infrastructure, 100 vehicular, and 100 smartphone user sources. Reported event timestamps were included in the report messages. We observed longer processing times for decentralized implementation using SCs. However, computational times cannot be considered realistic due to the simulated blockchain environment provided by Ganache. On the other hand, events were pushed continuously, even though they represent a simulated period of one month. A limit of a minimum of five reports per event was set to ensure that we obtain a sufficient number of reports to perform an event detection using weighted majority voting. Artificially generated delays in event reporting timestamps were used to represent more a realistic nature of the system (Table 2). On the other hand, such delays cause longer delays in the event detection phase.

Infrastructure sources were given the constant reputation (weight) of 1, as they are expected to be frequently calibrated. The initial set of vehicles was designed with 80% of correct reporting. Users were categorized into three categories—50% of users were totally honest and accurate, 20% were designed to be misreporters, and the remaining 30% were assigned an initial accuracy ratio of 0.8. The average correctness rate was obtained using weighted majority voting, which uses source reputation values as weights of 0.76.

Assessing sources’ reputation is crucial for more effective event detection and cleaning-up of misreporters’ data. Despite the quite simple mechanism that was used, we were able to detect all of of the users that were meant to misreport on every event they observed. Infrastructure sources, on the other hand, were given a constant reputation value that cannot be changed. Most data sources (vehicles and users) fall in-between the two extremes. Assessed reputation value for more than 90% (163) of the remaining data sources lies in the two 10%-bins surrounding the initial reporting accuracy ratio value as seen in Table 3.

The same application that was used in the driving simulator for persona verification was used to gather real-life traffic reports from users. The obtained data set consisted mostly of congestion and roadworks reports. Due to heavy traffic during holidays and roadworks, some congestions and traffic jams were several kilometers long. As our solution mostly focuses on events like accidents and dangerous driving conditions, the location-based aggregation module groups events in a rather small radius. Thus, a long-lasting traffic jam can be detected as several separate events. Moreover, some users report both roadworks and traffic congestion, the latter being caused by the former.

## 5. Conclusions and Future Work

The proposed event detection and source reputation assessment mechanism was designed with decentralized implementation in mind. By implementing it using a centralized relational database-based system and in a decentralized manner on the Ethereum blockchain, we showed that the proposed solution is equally viable in both environments.

Even though we did not provide a full crowdsourcing solution with included incentivizing mechanisms, the source reputation score recalculated after every report and can be directly used as a base for rewarding the users. Therefore, various incentivizing mechanisms (social, monetary, and token based) and rewarding schemes (uniform, variable, and lottery) can be implemented on top of the proposed solution. The Ethereum ecosystem also provides an environment for issuing tokens, which is a good fit for incentivizing and rewarding schemes.

Despite addressing the trust issues related to closed and centralized systems, blockchain implementation also exhibits several drawbacks. The first, and probably the most important one, is the required determinism of algorithms used, if implementing the whole event detection system using SCs. Regarding data storage and options, both the relational database- and blockchain-based implementation offer temporary, as well as permanent, storage. Considering storage expenses on the blockchain, local scope memory storage is preferred to the more expensive permanent storage. Therefore, during processing, data is stored in a temporary SC memory storage, while results are permanently stored on the blockchain. Even though relying on SC logic adds complexity and computational delays to the event detection process, the choice of not permanently storing event reports provides numerous advantages over the most straightforward choice; that is, storing event reports on the blockchain and outsourcing the analysis to the oracles or services outside of the blockchain.

The blockchain also guarantees consensus between entities that do not necessarily trust each other. However, malicious users can organize in misreporting groups to intentionally attack the system. To counter that, fraud detection mechanisms can be used, regardless of implementation. Moreover, in case of non-deterministic or computationally expensive methods, fraud detection and analysis can also be implemented using off-chain oracles—external service or data providers.

Due to human mobility, some exceptionally large time differences between the first and the fifth reports were observed in sparse traffic scenarios. Streaming-like processing mechanisms should be implemented to overcome this problem in real-life implementation. While the Boyer-Moore majority voting algorithm can be implemented in such a way in centralized approach, it could cause huge rises of storage and computational costs in blockchain-based systems due to more frequent state changes.

Instead, local off-chain event detection and reputation assessment processing could be implemented in relatively long-lived vehicular social networks that occur inside convoys and fleets of vehicles travelling the same direction. This will lower computational and storage costs and minimize processing times, while at the same time maintain the globally accessible event and reputation database.

As various kinds of traffic events and offences are not objectively measurable, the proposed solution was designed to support participatory mobile crowdsensing data gathering. Relying on users, in addition to infrastructure- and vehicle-originating traffic event reports, we add some degree of uncertainty into the system. Depending on the environment, observers’ personality, previous experiences with and in traffic, their current mood, the reason they are on the road, etc., the perception of a traffic event can differ greatly from person to person. These human characteristics provide some level of uncertainty at the very beginning of the crowdsensing system.

As pointed out in real-life experiments, in case of traffic jams caused by roadworks, users report both kinds of events. In the future, similar and consequential events should be treated accordingly—e.g., using a probability matrix to model user reporting behavior and event causality. In the future, we plan to improve the event detection mechanism by incorporating a user-reporting model. In addition to already presented personas, we plan to model the typical user reporting preferences. As such experiments cannot be done in real-life situations, a simulation environment such as one used in this study will be used.

## Figures and Tables

**Table 1:** Excerpt from the sources/personas table.

ID	Source Type	Reporting Ratio	Apparent Accuracy Aatio	Notes
34	Vehicle	0.8	1	
140	User	0.8	1	Reporting near school
156	User	0.5	0.8	Reporting dangerous situations
446	Vehicle	0.5	1	
461	Government	1	1	Police
510	User	0.7	0.8	Sometimes wrong reports
527	User	0.6	0.0	Misreporter

**Table 2:** Example of event reports, and the detected event.

**Event**	**Timestamp**	**Event Type**	**Location**	
	4.09.2018 22:42	Careless Driving	46.0645718, 14.5066889	
**Reports**	**Timestamp**	**Reported event type**	**Location**	**Source type**
	4.09.2018 22:42	careless driving	46.0678532, 14.5088111	user
	4.09.2018 22:43	careless driving	46.0679797, 14.5086931	user
	4.09.2018 22:44	careless driving	46.0677266, 14.508618	user
	4.09.2018 22:42	red light	46.0673842, 14.5086394	user
	4.09.2018 22:42	speeding	46.0665059, 14.5077382	vehicle
**Detected event**	**Timestamp**	**Event type**	**Location**	**Certainty**
	4.09.2018 22:42	careless driving	46.067490, 14.508500	0.6

**Table 3:** Source reputation value assessment correctness.

Data Source	Infrastructure	Users			Vehicles
		Misreporting	Accurate	In-between	
**Number of sources**	100	20	50	30	100
**Initial accuracy ratio**	1	0	1	0.8	0.8
**Assessed reputation correctness**	Not assessed	100%	>90% of sources in range [0.7,0.9]		
